# Supplementary material for: A scalable Li-Al-Cl stratified structure for stable all-solid-state lithium metal batteries
Source: Nat Commun. 2024 May 17;15:4202. doi: 10.1038/s41467-024-48585-7 (PMC11101657; doi:10.1038/s41467-024-48585-7)
Supplement: Supplementary file 1 — Supplementary Information [file 41467_2024_48585_MOESM1_ESM.pdf]

SUPPLEMENTARY INFORMATION

A scalable Li-Al-Cl stratified structure for stable all-solid-state lithium metal batteries

Han Su[1], Jingru Li[1], Yu Zhong[1,*], Yu Liu[1], Xuhong Gao[1], Juner Kuang[1], Minkang Wang[1], Chunxi Lin[1], Xiuli Wang[1], Jiangping Tu[1,*]

[1] State Key Laboratory of Silicon and Advanced Semiconductor Materials, Key Laboratory of Advanced Materials and Applications for Batteries of Zhejiang Province, School of Materials Science and Engineering, Zhejiang University, Hangzhou 310027, China

These authors contributed equally: Han Su, Jingru Li

[*] Corresponding authors

Email: yu_zhong@zju.edu.cn; tujp@zju.edu.cn

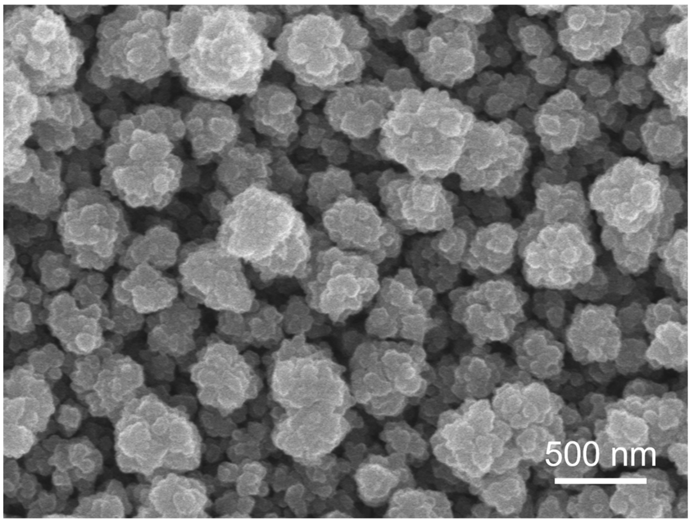

Supplementary Fig. 1 Magnified top-view SEM image of the $AlCl_3$-deposited Li.

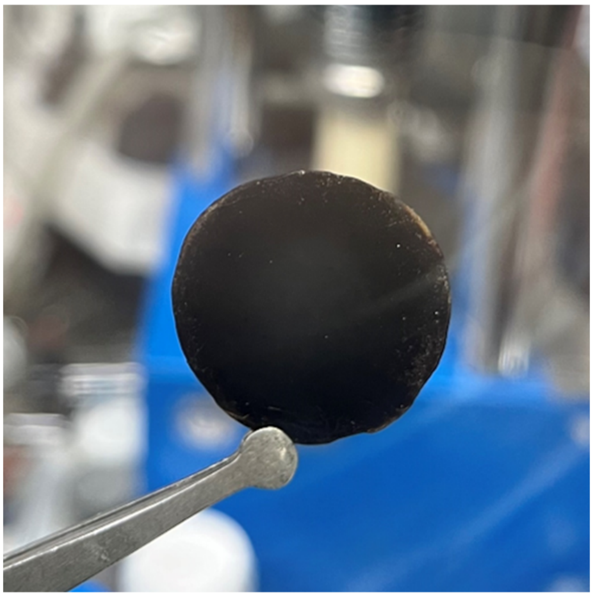

Supplementary Fig. 2 Optical image of the $AlCl_3$-deposited Li.

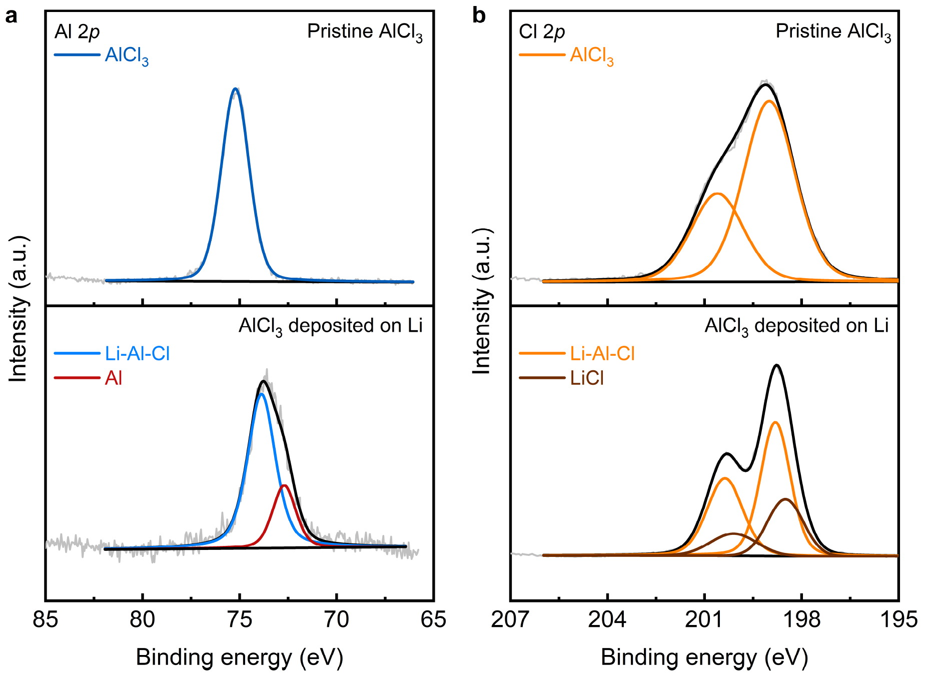

Supplementary Fig. 3 Compositional evolutions of $AlCl_3$ before and after its deposition on Li metal. **a, b** Al 2*p* (**a**) and Cl 2*p* (**b**) XPS spectra of pristine $AlCl_3$ powder and the $AlCl_3$ deposited on Li.

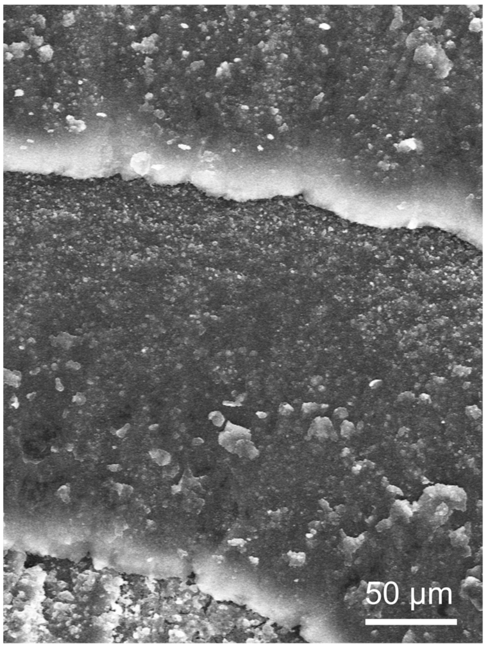

Supplementary Fig. 4 Cross-sectional SEM image of the $AlCl_3$-deposited Li after winding.

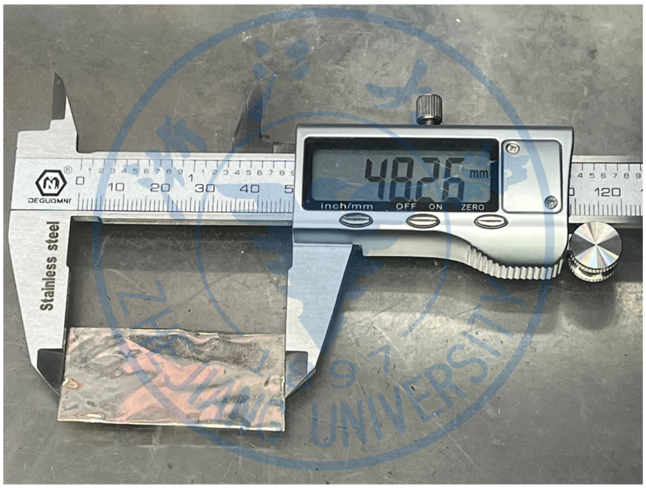

Supplementary Fig. 5 Optical image of the large-sized LACSS.

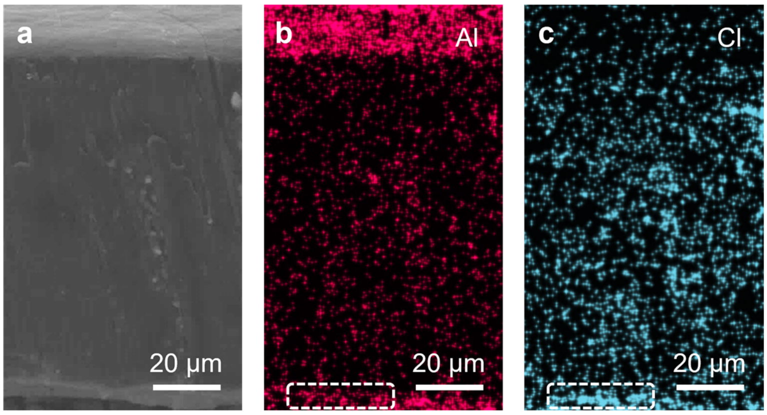

Supplementary Fig. 6 Cross-sectional SEM observation of the LACSS with a thickness of ~ 100 μm. **a** Cross-sectional SEM image of the LACSS with a thickness of ~100 μm. **b, c** Corresponding EDS mapping results of Al (**b**) and Cl (**c**). The thickness of this LACSS is different from that of the LACSS presented in Fig. 1c.

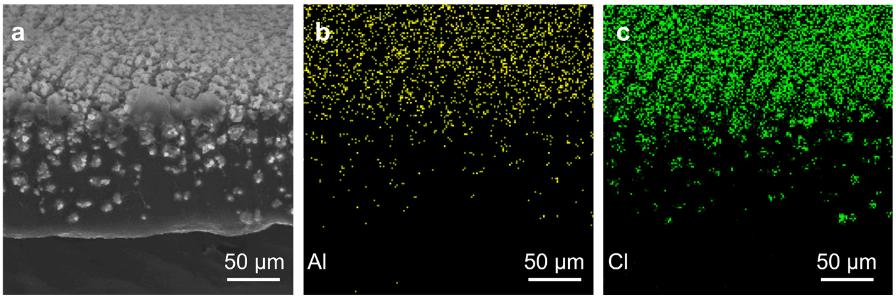

Supplementary Fig. 7 Cross-sectional SEM observation of the $AlCl_3$-deposited Li foil after rolling. **a** Cross-sectional SEM image of the $AlCl_3$-deposited Li foil after rolling. **b, c** Corresponding EDS mapping results of Al (**b**) and Cl (**c**).

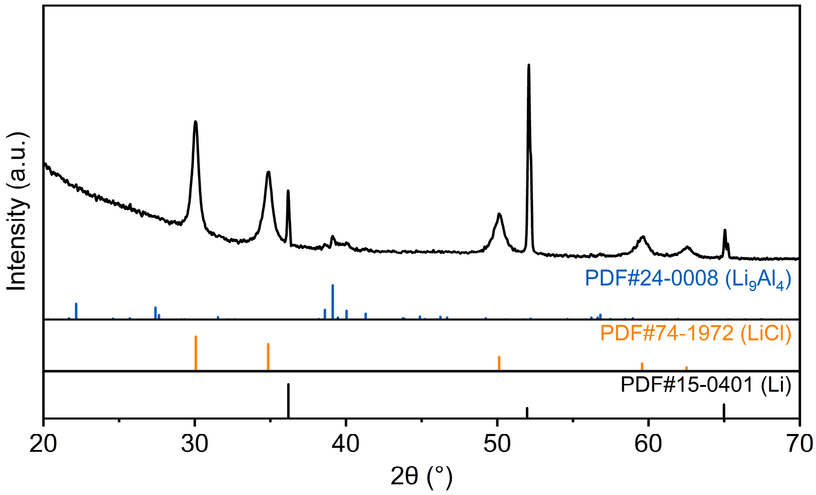

Supplementary Fig. 8 XRD pattern of the LACSS prepared by extending the exposure time of fresh Li to the $AlCl_3$ gas.

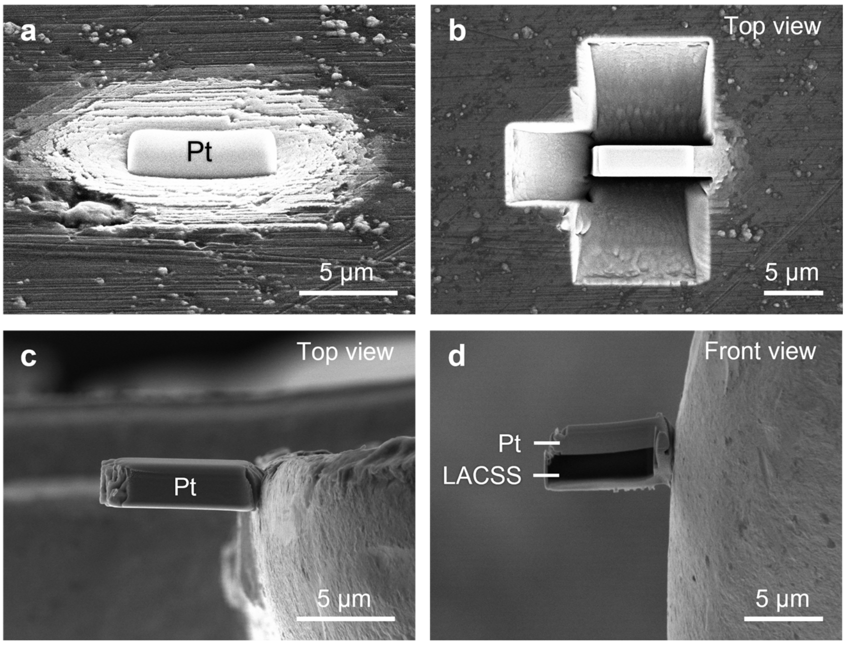

Supplementary Fig. 9 Preparation process of the LACSS lamella for TEM characterization via FIB-SEM. **a** SEM image of the deposited Pt layer on the surface of LACSS at 25 °C. **b** Top-view SEM image of the etched LACSS around the deposited Pt layer via focused Ga ion beam at −195 °C. **c, d** Top-view (**c**) and front-view (**d**) SEM images of the lamella lifted out and weld to the Cu grid via a manipulator arm at −195 °C.

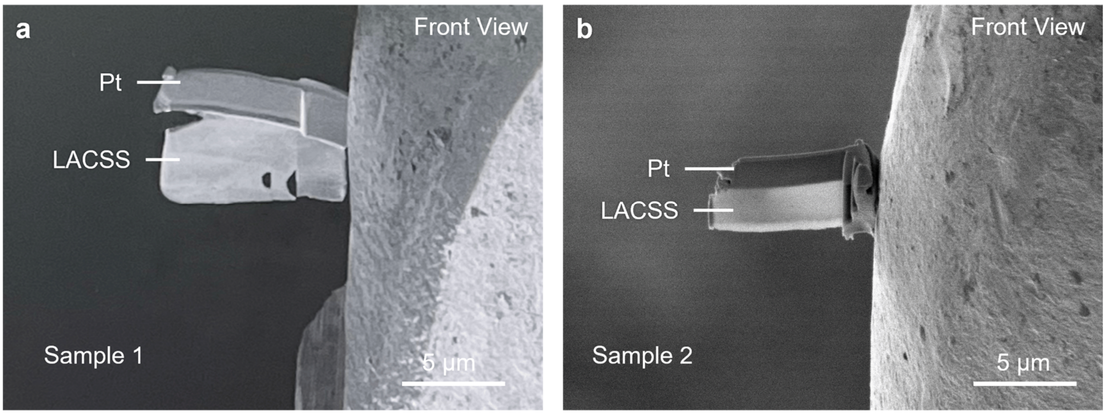

Supplementary Fig. 10 Front-view SEM images of LACSS lamellas for TEM observation. **a** SEM image of the lamella detaching from the Pt layer after thinning. **b** SEM image of a relatively thick lamella maintaining a tight connection with Pt layer after thinning.

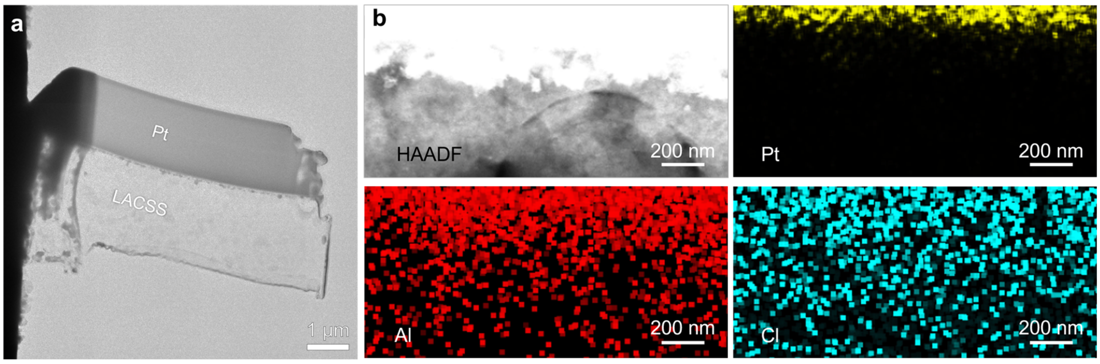

Supplementary Fig. 11 Characterizations of the lamella displayed in Supplementary Fig. 10b via TEM. **a** TEM image of the relatively thick lamella. **b** Magnified HAADF-TEM image of the lamella and corresponding EDS mapping results of Pt, Al and Cl.

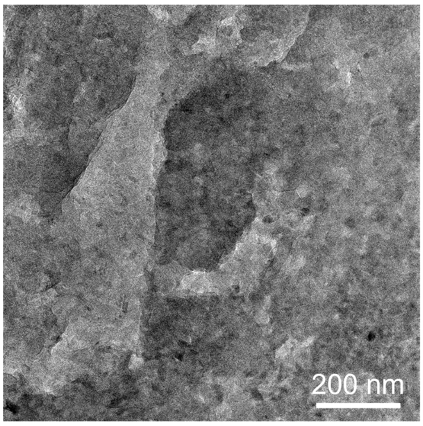

Supplementary Fig. 12 TEM image of the LACSS lamella at low magnification.

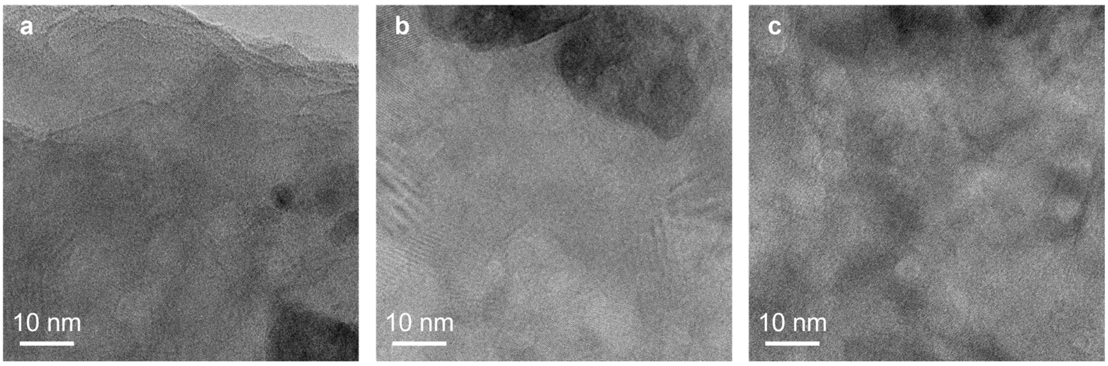

Supplementary Fig. 13 HR-TEM images of the selected regions 1-3 in Fig. 2e. **a** Region 1. **b** Region 2. **c** Region 3.

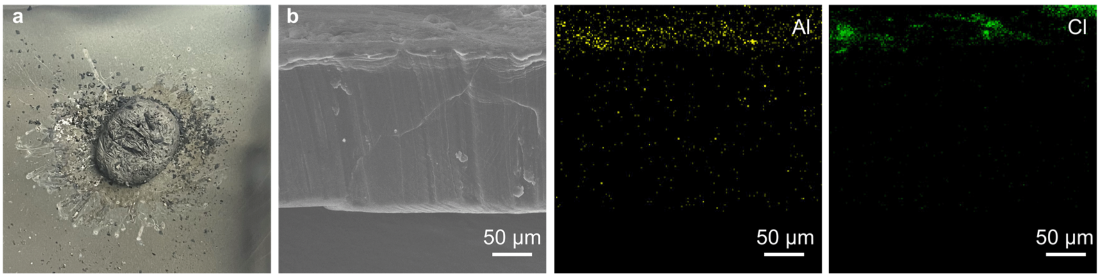

Supplementary Fig. 14 $AlCl_3$/Li complex prepared via dispersing $AlCl_3$ into molten Li. **a** Optical image of the melting $AlCl_3$/Li complex with a mass ratio of 1: 10 at 350 °C. **b** Cross-sectional SEM image of the rolled post-reaction products of $AlCl_3$/Li complex, along with corresponding EDS mapping results of Al and Cl. The post-reaction products of the $AlCl_3$/Li complex for cross-sectional SEM characterization were prepared by rolling the complex after cooling down. The EDS mapping result indicates that Al and Cl congregate on the surface of the complex.

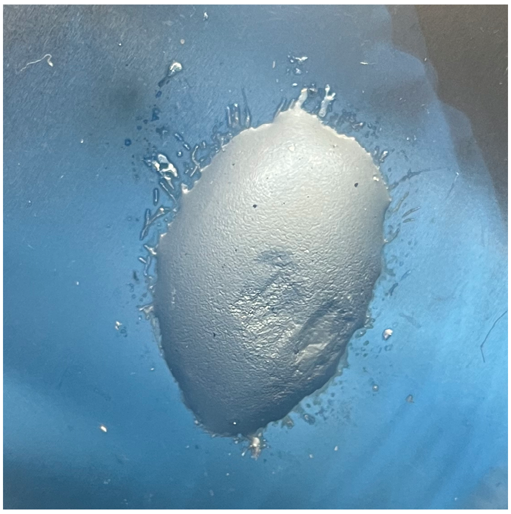

Supplementary Fig. 15 Optical image of the Li_9Al_4/Li complex after melting and cooling. The Li_9Al_4/Li complex was fabricated by melting Li and Al at a high temperature of 400 °C in an atomic ratio of 98.1:1.9. After a continuous stirring process for 30 min, the surface turned grey. After cooling down, a layer of grey-color particles covered the surface of the Li_9Al_4/Li complex.

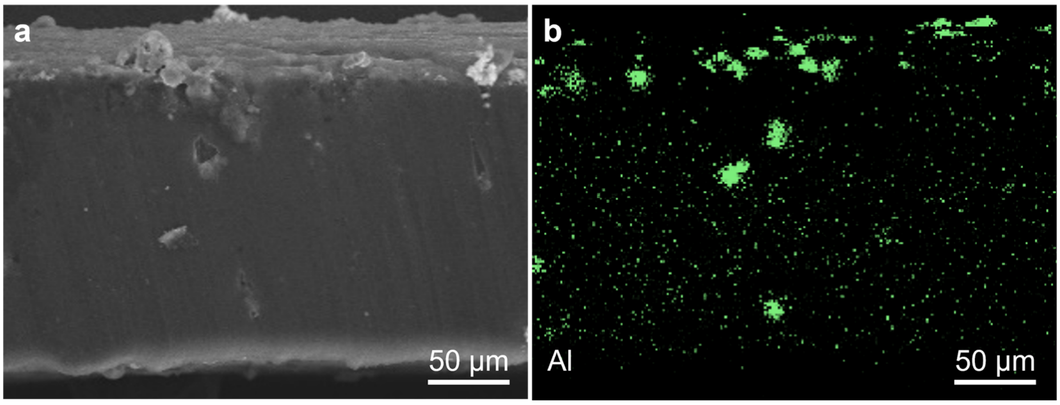

Supplementary Fig. 16 Cross sectional SEM observation of the Li_9Al_4/Li complex. **a** Cross-sectional SEM image of the Li_9Al_4/Li complex. **b** EDS mapping result of Al. The Li_9Al_4/Li foil for cross-sectional SEM characterization was prepared by rolling the complex after melting and cooling. The EDS mapping result indicates that the Al-rich phase tends to float on the surface of the Li_9Al_4/Li foil.

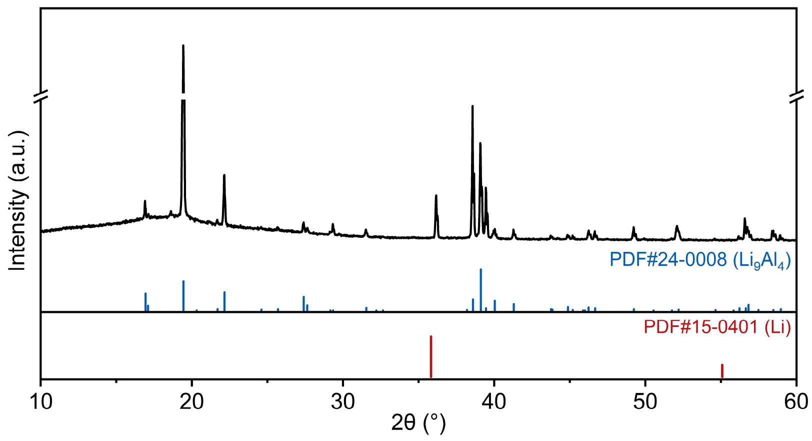

Supplementary Fig. 17 XRD pattern of the Li_9Al_4/Li foil. The XRD pattern shows a higher ratio of the characteristic peaks of Li_9Al_4 than those of Li. Based on this, it can be inferred that the surface of Li_9Al_4/Li foil contains more Li_9Al_4 phase than the bulk.

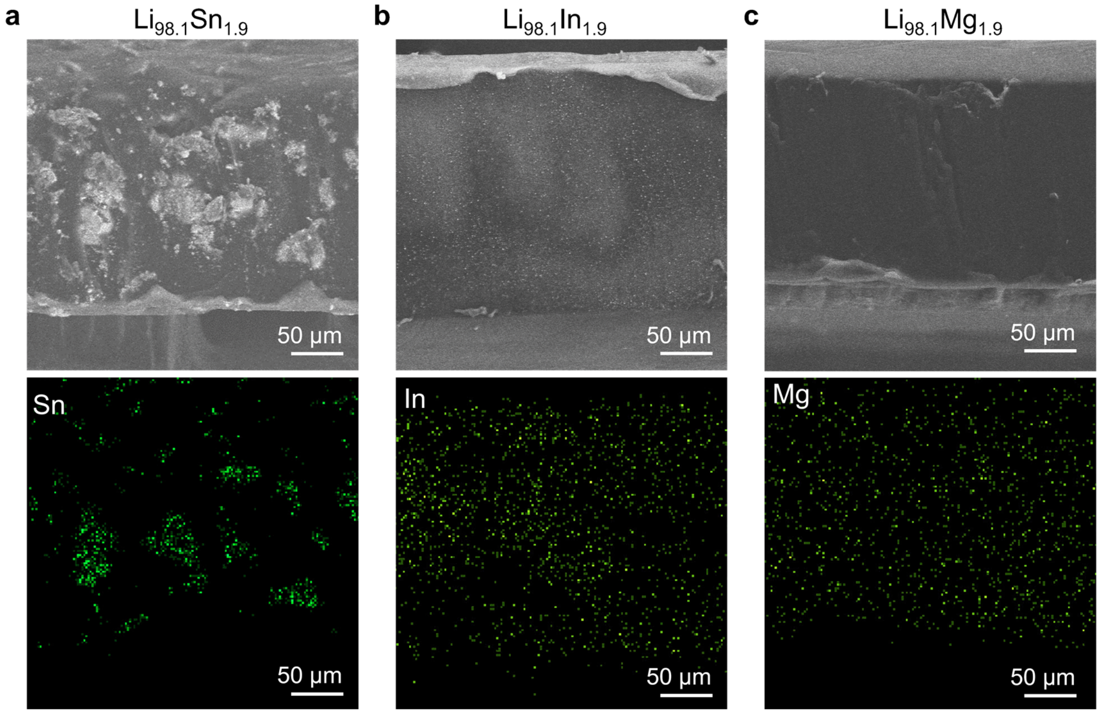

Supplementary Fig. 18 Characterization of other Li alloys (with cubic structure)/Li complexes. **a** Cross-sectional SEM image of the Li-Sn/Li complex prepared by melting-cooling and the corresponding EDS mapping result of Sn. **b** Cross-sectional SEM image of the Li-In/Li complex prepared by melting-cooling and the corresponding EDS mapping result of In. **c** Cross-sectional SEM image of the Li-Mg/Li complex prepared by melting-cooling and the corresponding EDS mapping result of Mg. The Li alloys/Li complexes were prepared by melting Li with alloy elements in an atomic ratio of 98.1:1.9 at 400 °C first and rolling the post-reaction products after cooling down.

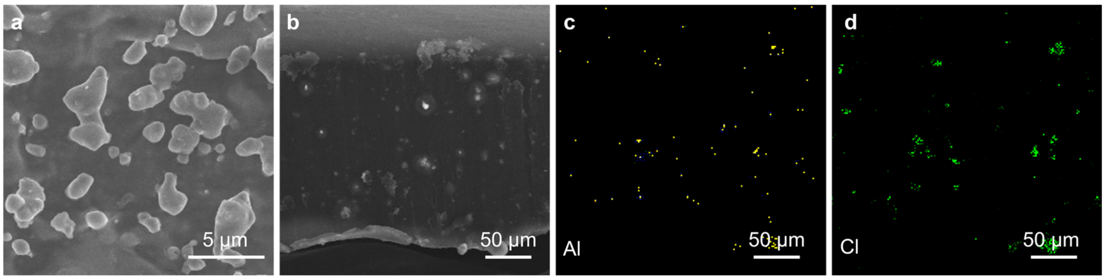

Supplementary Fig. 19 Investigation of the size effect in solid-state phase separation. **a** SEM image of hand-milled $AlCl_3$ particles. **b, c, d** Cross-sectional SEM image of the $AlCl_3$-Li foil (**b**) and EDS mapping results of Al (**c**) and Cl (**d**). Pristine $AlCl_3$ powder was hand-milled for 20 min to reduce the particle size. However, the average size of the milled $AlCl_3$ was still larger than 1 µm. The $AlCl_3$-Li foil was prepared by spreading the hand-milled $AlCl_3$ on the surface of a polished Li metal first, and heating them together at 178 °C, which was followed by a winding-rolling process.

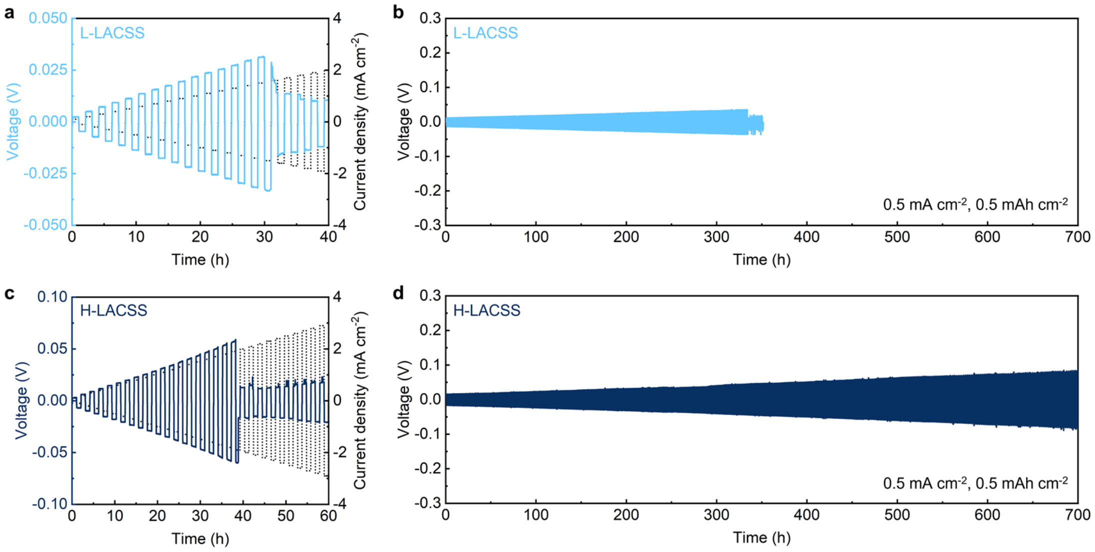

Supplementary Fig. 20 Electrochemical performance of L-LACSS and H-LACSS symmetric cells**. a** Voltage profiles of the L-LACSS symmetric cell during step-increased current density tests. **b** Galvanostatic cycling performance of the L-LACSS symmetric cell at 0.5 mA cm^{-2}/0.5 mAh cm^{-2}. **c** Voltage profiles of the H-LACSS symmetric cell during step-increased current density tests. **d** Galvanostatic cycling performance of the H-LACSS symmetric cell at 0.5 mA cm^{-2}/0.5 mAh cm^{-2}.

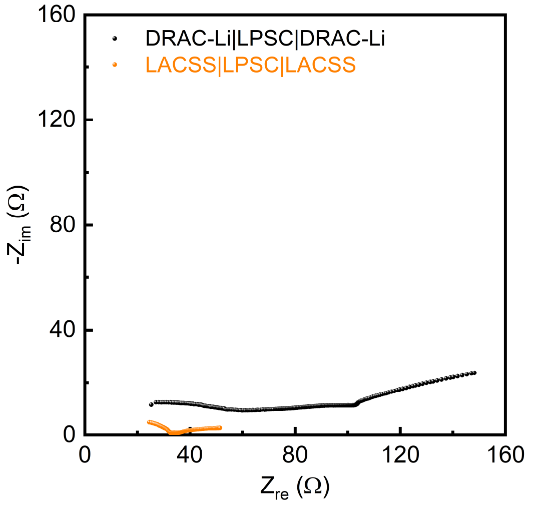

Supplementary Fig. 21 EIS spectra of symmetric cells using the directly rolled $AlCl_3$-deposited Li (DRAC-Li) and the LACSS as the electrode before cycling.

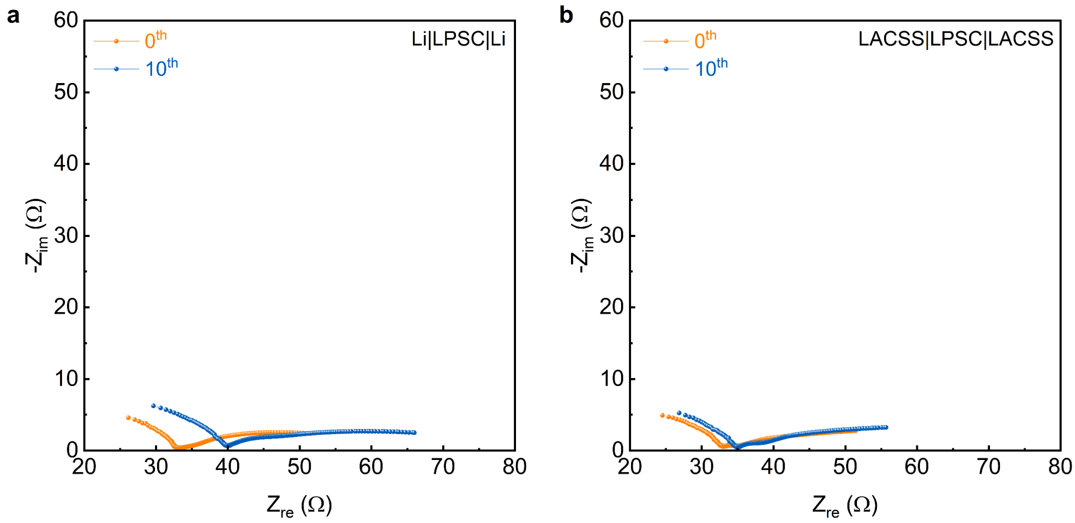

Supplementary Fig. 22 EIS spectra of the bare Li symmetric cell and the LACSS symmetric cell before and after cycling at 0.5 mA cm^{-2}/0.5 mAh cm^{-2} for 10 cycles. **a** EIS spectra of the bare Li symmetric cell before and after cycling at 0.5 mA cm^{-2}/0.5 mAh cm^{-2} for 10 cycles. **b** EIS spectra of the LACSS symmetric cell before and after cycling at 0.5 mA cm^{-2}/0.5 mAh cm^{-2} for 10 cycles.

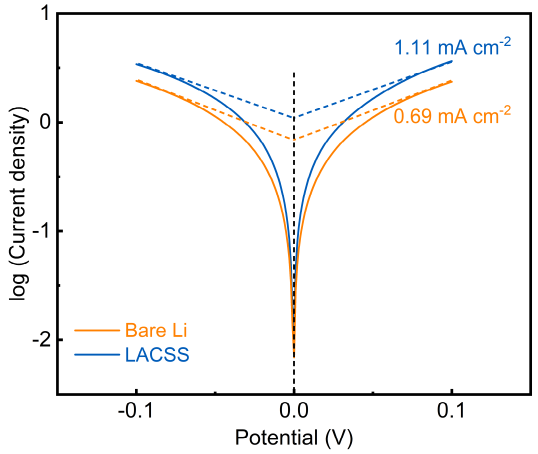

Supplementary Fig. 23 Tafel plots of the bare Li symmetric cell and the LACSS symmetric cell after 10 cycles at 0.5 mA cm^{-2}/0.5 mAh cm^{-2} obtained by LSV.

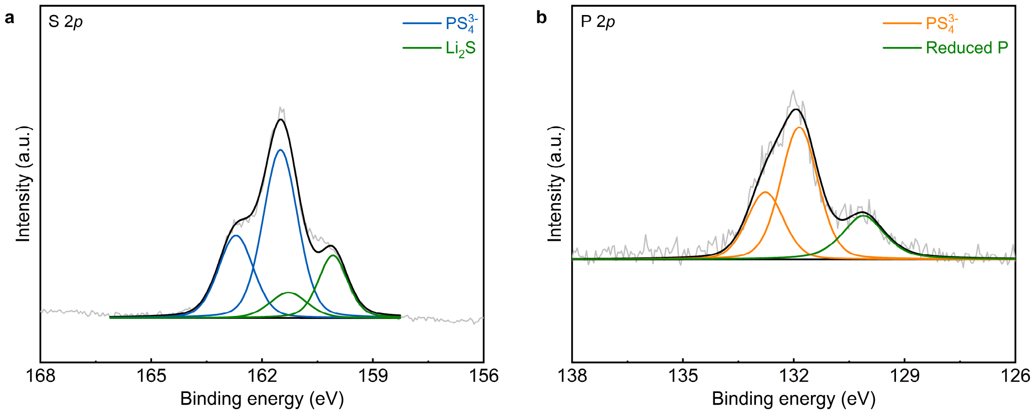

Supplementary Fig. 24 XPS spectra of the bare Li/LPSC interface after cycling. **a** S 2*p*. **b** P 2*p*.

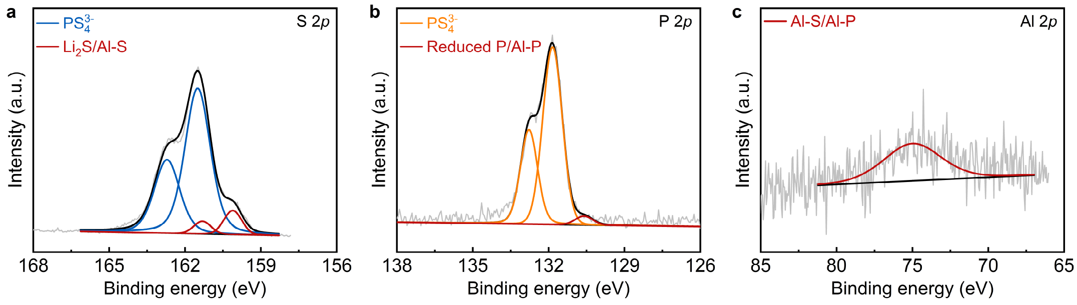

Supplementary Fig. 25 XPS spectra of the LACSS/LPSC interface after cycling. **a** S 2*p*. **b** P 2*p*. **c** Al 2*p*.

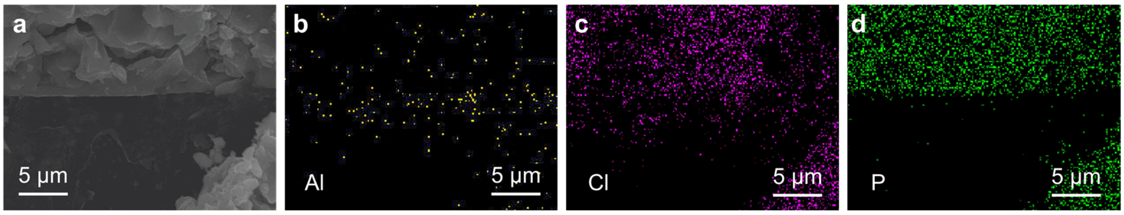

Supplementary Fig. 26 Cross-sectional SEM observation of the LACSS/LPSC interface after the deposition of 2 mAh cm^{-2} Li. **a** Cross-sectional SEM image of the LACSS/LPSC interface. **b, c, d** Corresponding EDS mapping results of Al (**b**), Cl (**c**) and P (**d**).

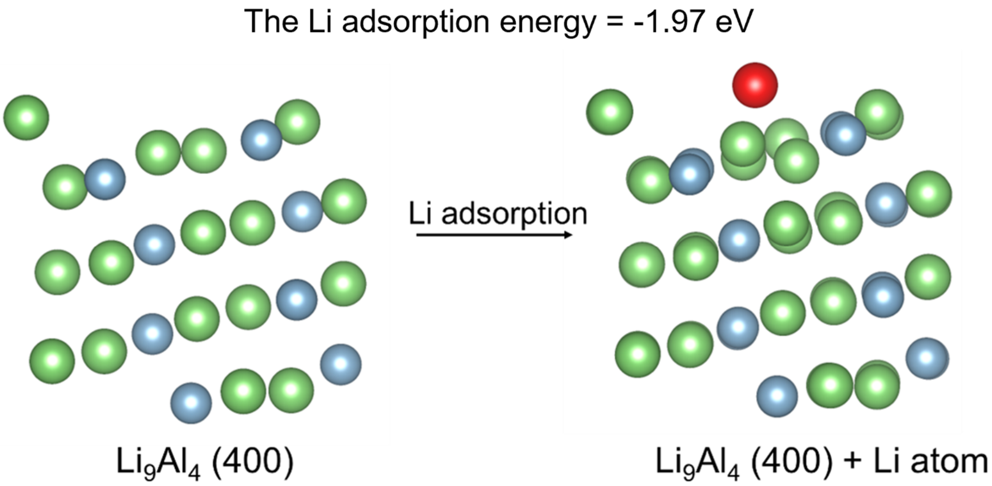

Supplementary Fig. 27 Adsorption of Li atom on the Li_9Al_4 (400) surface. The green balls represent Li atoms in the Li_9Al_4, the red ball represents the Li atom adsorbed on the Li_9Al_4 surface and the blue balls represent Al atoms. The adsorption energy is calculated to be −1.97 eV.

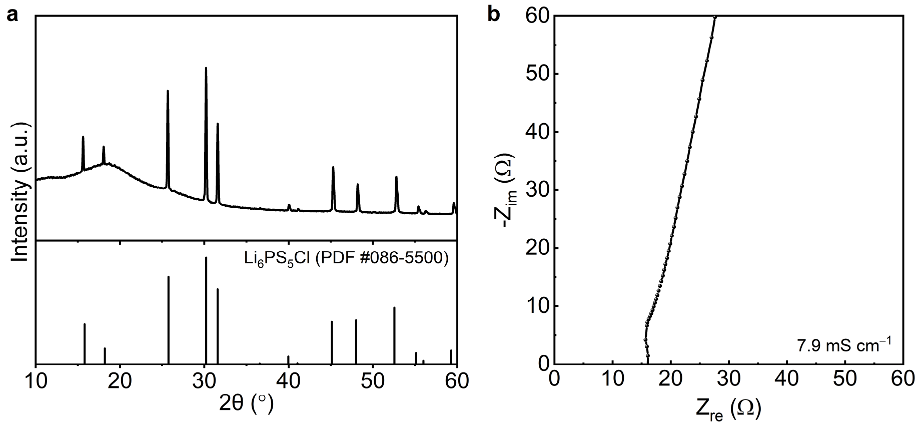

Supplementary Fig. 28 Characterizations of the LPSC electrolyte. **a** XRD pattern of the LPSC powder. **b** Nyquist plot of the carbon | LPSC | carbon block cell at 25 °C. The ionic conductivity of the LPSC is determined to be 7.9 mS cm^{-1}.

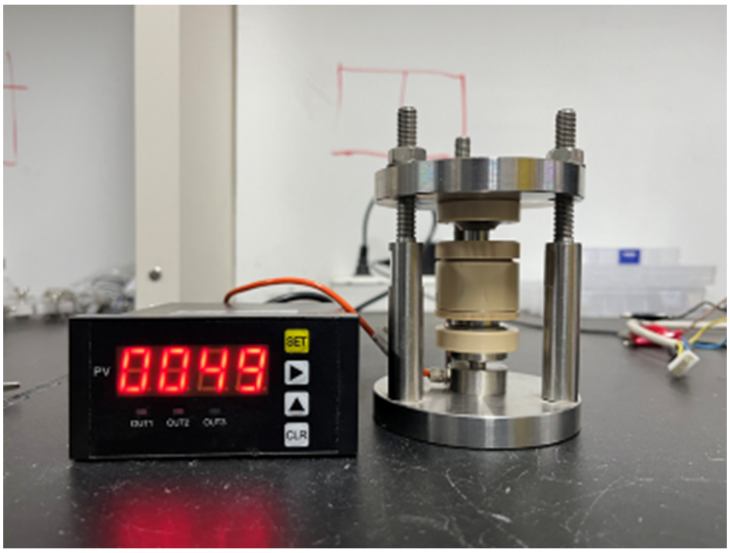

Supplementary Fig. 29 Measured force on the testing full cell. Based on this, the applied pressure on the cell is calculated to be 6 MPa.

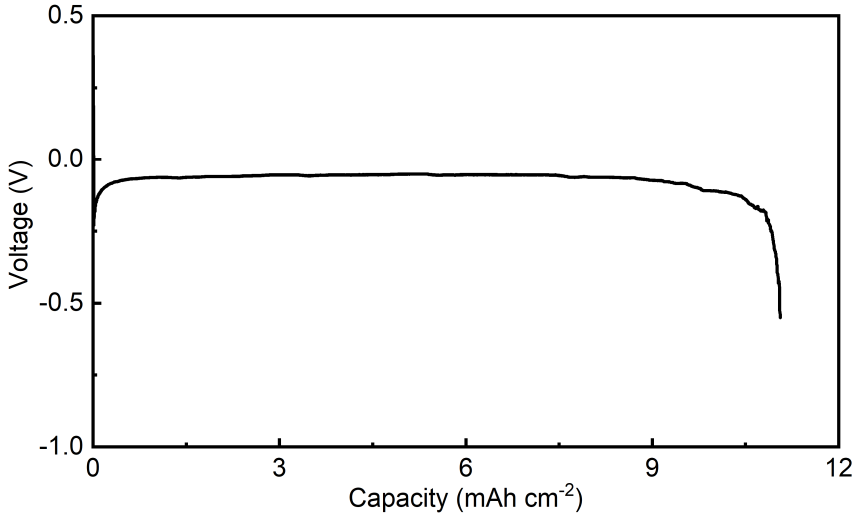

Supplementary Fig. 30 Li stripping curve of the 60 μm-thick LACSS till −0.5 V in the LACSS || Cu coin cell. Liquid electrolyte (1 M $LiPF_6$ in EC:DEC (1:1 in volume) with 5 wt% FEC) was applied due to its better wettability towards electrodes, which allowed the full release of the electrode's capacity.

Supplementary Table 1. DFT calculation results of the surface energy of LiCl (γ_{LiCl}), the LiCl/Li interface energy ($\gamma_{LiCl/Li}$) and the value of γ_{LiCl}-$\gamma_{LiCl/Li}$.

γ_{LiCl} (J m^{-2})	$\gamma_{LiCl/Li}$ (J m^{-2})	γ_{LiCl}-$\gamma_{LiCl/Li}$ (J m^{-2})
0.094	0.514	−0.42
